# Challenges of dengue and coronavirus disease 2019 coinfection: two case reports

**DOI:** 10.1186/s13256-021-02973-5

**Published:** 2021-08-30

**Authors:** Olga Lucia Agudelo Rojas, María Elena Tello-Cajiao, Fernando Rosso

**Affiliations:** 1 Fundación Valle del Lili, Centro de Investigaciones Clinicas (CIC), Cali, Colombia; 2 Internal Medicine Department, Infectious Disease Service, Fundación Valle del Lili, Cali, Valle del Cauca Colombia

**Keywords:** Dengue, COVID-19, Coinfection, SARS CoV-2

## Abstract

**Background:**

Dengue fever and coronavirus disease 2019 have now begun to overlap within tropical and subtropical regions. This is due to the high prevalence of dengue fever in these regions and the current severe acute respiratory syndrome coronavirus 2 pandemic situation. The similarity of symptoms between the two diseases can confuse diagnoses, but coinfection can also occur.

**Case presentation:**

We present two cases of patients with dengue and severe acute respiratory syndrome coronavirus 2 coinfection. The first case is that of a 24-year-old Hispanic woman with acute fever, odynophagia, and diarrhea, without respiratory symptoms and with positive molecular tests for both dengue and severe acute respiratory syndrome coronavirus 2. The second case is that of a 59-year-old Hispanic male patient with fever and respiratory symptoms of 2 weeks duration, negative molecular tests, and positive serological tests for both viruses. The clinical and epidemiological characteristics of both viral infections can help elucidate diagnoses and prognoses.

**Conclusions:**

Severe dengue infection is common in young adults, while coronavirus disease 2019 is generally asymptomatic. In older people, the severity of dengue fever will depend on their comorbidities or the infectious serotype, but coronavirus disease 2019 is consistently more severe in this group. The accurate diagnosis of both infections can better guide clinical management, as well as public health actions in transmission control, now especially important during the coronavirus disease 2019 pandemic.

## Background

Dengue fever is an endemic arbovirus in tropical and subtropical regions [1]. From 2019 to the present, it has exhibited epidemic behavior worldwide, with approximately 4.2 million cases reported in 2019. In the Americas alone, 3.1 million cases were reported last year, with more than 25,000 classified as severe [2]. On the other hand, severe acute respiratory syndrome coronavirus 2 (SARS CoV-2), which causes coronavirus disease 2019 (COVID-19), was declared a pandemic in March 2020 by the World Health Organization (WHO). It currently has infected approximately 44 million worldwide, and caused more than 1 million deaths [3]. As expected, in 2020, dengue fever and COVID-19 began to overlap, and several dengue-endemic countries, such as Singapore, Thailand, India, and Bangladesh, have already reported coinfection [4–7].

This situation represents a challenge in the diagnosis and treatment of both infections, given the differences in the transmission mechanisms and their management implications. It is a priority to establish a timely diagnosis of COVID-19 in areas that are also experiencing a dengue epidemic to avoid delays in proper treatment [6, 8]. Therefore, laboratory tests play an important role in the clinical field and public health actions to control COVID-19 transmission. In this article, we present and analyze the cases of two patients with symptoms and laboratory findings compatible with dengue fever as well as COVID-19 who were treated in a health institution in Cali, Colombia, in June 2020.

## Case report 1

A 24-year-old Hispanic female health worker with no significant medical history presented with symptoms of 6 days duration consisting of fever, odynophagia, adynamia, myalgia, arthralgia, vomiting, and diarrhea. She had no respiratory symptoms. On admission, her vital signs were a blood pressure of 101/62 mmHg, heart rate of 92 beats per minute, respiratory rate of 17 breaths per minute, O_2_ saturation of 92% (room air), and temperature of 37.4 °C. Physical examination showed dry oral and conjunctival mucosa, with no other findings.

**Fig. 1:**
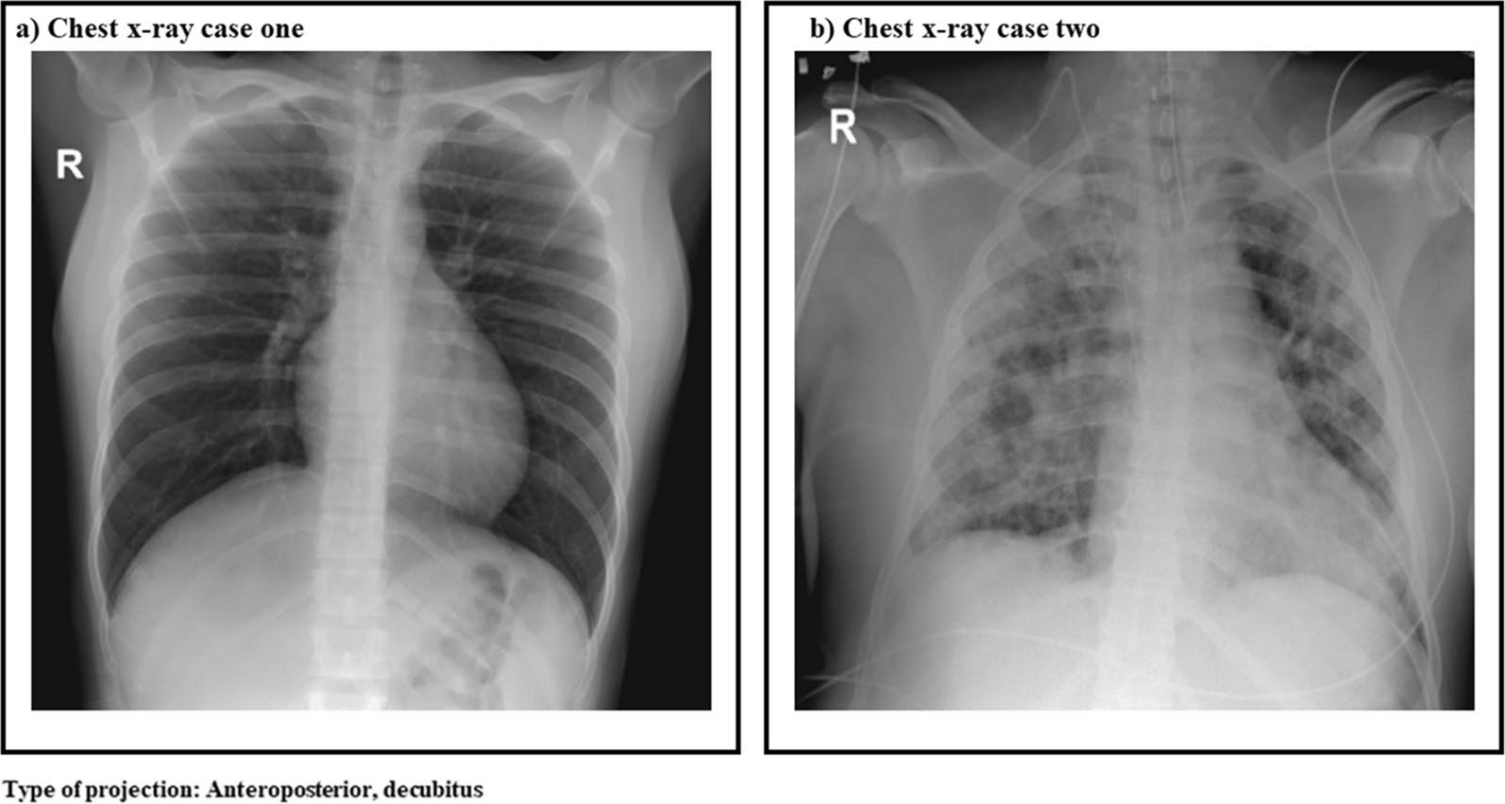
X-ray images of each case

**Fig. 2:**
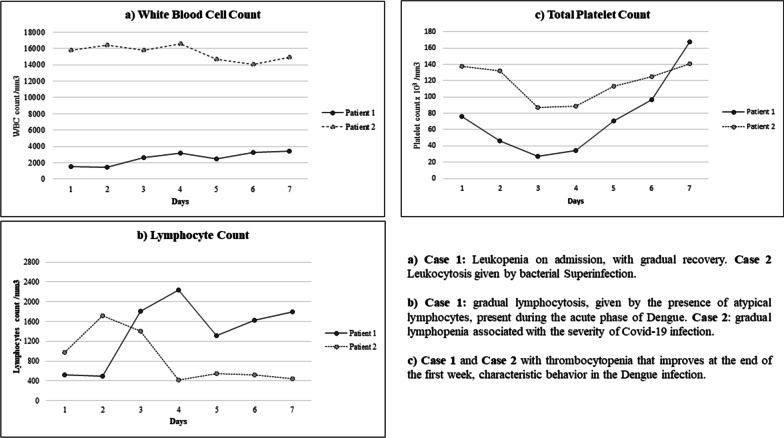
Behavior of leukocytes and platelets during coinfection

On initial laboratory screening, the patient demonstrated thrombocytopenia (platelets 76 × 10^3^/ml) and leukopenia [white blood cells (WBC) 1560 cells/ml, 57.8% neutrophils, 33.4% lymphocytes] without hemoconcentration. Transaminases [aspartate aminotransferase (AST) 666 IU and alanine aminotransferase (ALT) 516 IU], and d-dimer [0.648 µg/ml (normal value until 0.5 µg/ml)], were elevated, and dengue immunoglobulin M (IgM)/nonstructural protein 1 (NS1) and dengue reverse transcription polymerase chain reaction (RT-PCR) were positive. Because of this, dengue fever with warning signs was diagnosed, and she was admitted to the intensive care unit (ICU) for close monitoring. On the second day of stay, the patient presented a worsening of vomiting, in addition to dysgeusia. Considering her occupational exposure to COVID-19 cases, SARS CoV-2 the real-time RT-PCR (rRT-PCR) was conducted, which was positive. Chest X-ray did not show any abnormalities (Fig. 1a). With these findings, coinfection was confirmed. Details of the coinfection clinical description are presented in Table 1. The patient was kept under continuous surveillance in the ICU, as her laboratory test results revealed worsening thrombocytopenia and leukopenia over the days and gradual atypical lymphocytosis (Fig. 2). However, she did not exhibit signs of active bleeding. She was discharged after 6 days of hospitalization. She was followed up by telephone, and she reported adequate resolution of her symptoms. The last follow-up was done in July 2020.

**Table 1:** Clinical description of coinfection cases and their outcomes

	Case 1	Case 2
Admission date	14 June 2020	19 June 2020
Days of symptoms on admission	6	20
Admission diagnosis	Dengue fever	COVID-19
Day 1 hospitalization	Dengue IgM/NS1 positive (6 days of evolution) rRT-PCR dengue positive First chest X-ray clean	SARS CoV-2 rRT-PCR negative (20 days of evolution) First chest X-ray: extensive interstitial infiltrates in both lung fields, blood culture negative
Day 2 hospitalization	SARS CoV-2 rRT-PCR positive (8 days of evolution)	Dengue IgM/IgG positive (22 days evolution) RT-PCR dengue negative
Day 3 hospitalization	Second chest X-ray clean (9 days of evolution)	SARS CoV-2 rRT-PCR negative (23 days of evolution) Culture orotracheal secretion positive for *Staphylococcus aureus*
Day 4 hospitalization	–	SARS CoV-2 rRT-PCR negative (24 days of evolution)
Day 5 hospitalization	–	IgG SARS-CoV-2 positive (24-day evolution) Dengue IgM/IgG positive (24 days evolution)
Summary of clinical evolution and outcomes	The patient was kept under surveillance in the intensive care unit for having severe dengue criteria and coinfection with SARS CoV-2. She was discharged on the sixth day of hospitalization She was followed up by phone. She has had adequate evolution and has remained without symptoms. The last follow-up was done in July 2020	The patient was admitted with a diagnosis of severe COVID-19 pneumonia and dengue fever with alarm signs. He remained in intensive care for 2 months, with progressive clinical deterioration worsened by the development of *Klebsiella pneumoniae* sepsis. He required prolonged ventilatory support until he finally died
Final diagnosis	Dengue/SARS CoV-2 coinfection	Dengue/SARS CoV-2 coinfection

*IgM* immunoglobulin M, *IgG* immunoglobulin G IgG, *NS1* nonstructural protein 1, *rRT-PCR* real time reverse transcriptase-polymerase chain reaction

## Case report 2

A 59-year-old Hispanic male patient with a medical history of hypertension, obesity, and poorly controlled diabetes mellitus (last HbA1c was 13%) presented to the emergency department for fever of 20 days duration and worsening cough with expectoration, as well as progressive respiratory distress. On admission, his vital signs were a blood pressure of 144/83 mmHg, heart rate of 74 beats per minute, respiratory rate of 50 breaths per minute, O_2_ saturation of 40% (room air), and temperature of 38 °C. On physical examination, he was found to be febrile, somnolent, and polypneic, with marked respiratory effort. Emergency orotracheal intubation and transfer to the intensive care unit were necessary.

Initial screening showed thrombocytopenia (platelet count 138 × 103 cells/µl), leukocytosis (WBC 15,760 cells/ml, 90% neutrophils) with lymphopenia (6.20% lymphocytes), elevated transaminases (ALT 1507.4 IU and AST 3049.7 IU), elevated d-dimer (44,066 µg/ml), and negative RT-PCR SARS CoV-2 test. Chest X-ray showed extensive interstitial infiltrates in both lung fields (Fig. 1b). Considering the progressive decrease in total platelet count as well as gradual lymphopenia (Fig. 2), a dengue IgM/IgG test was conducted and was positive, while the dengue PCR-RT test was negative. According to radiographic findings and respiratory involvement, the RT-PCR test for SARS CoV-2 was repeated on two more occasions, with negative results. Therefore, IgG antibodies for SARS CoV-2 were assayed and were positive.

**Fig. 3:**
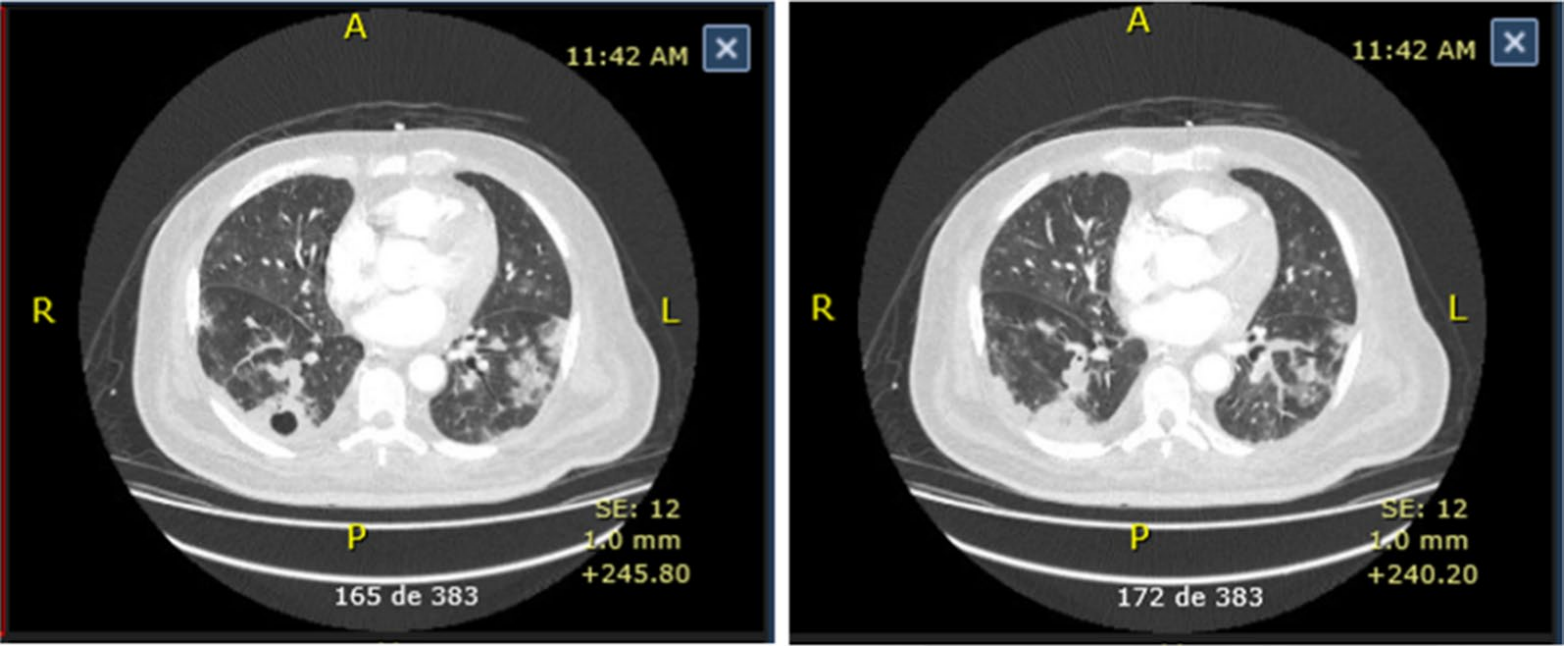
Computerized tomography angiography of the chest of case 2

Positive serology for both SARS CoV-2 and dengue virus confirmed coinfection, so it was considered that he had probably entered a late stage of the disease. Steroid management was indicated according to COVID-19 management guidelines, in addition to pronation cycles, to which he presented poor response and persistent hypoxemia. During his stay in intensive care, he presented multiple complications, such as acute pulmonary embolism, with signs of secondary thromboembolic pulmonary hypertension, bacterial superinfection in his lungs by *Klebsiella pneumoniae*, and Akin III acute renal failure. Computerized tomography angiography of the chest was performed, which confirmed acute pulmonary thromboembolism of multiple bilateral lobar and segmental bilateral branches with signs of secondary pulmonary hypertension and pulmonary changes suggestive of organizing pneumonia secondary to viral infection (Fig. 3). Details of the clinical description of the coinfection are presented in Table 1. The patient remained in the ICU for COVID-19, bacterial superinfection, and dengue with alarm signs for 63 days. His progression was slow, showing progressive clinical deterioration. He required prolonged mechanical ventilation and vasopressor management until he finally died.

## Discussion and conclusions

Physicians should be aware of the possibility of dengue and COVID-19 coinfection in areas with overlapping outbreaks and be aware of a potentially harmful interaction between these viruses. In the acute phases of both infections, symptoms are similar and overlap, which may delay appropriate diagnosis and treatment [7]. Details of the comparison between COVID-19 and dengue infection are presented in Table 2. This can have serious clinical consequences, either through failure of adequate hydration or delay in the use of anticoagulants, corticosteroids, and early mechanical ventilation [9, 10].

**Table 2:** Comparison between COVID 19–dengue

Clinical characteristics	COVID-19	Dengue
Cough	+	−
Fever	+	++
Dyspnea	+	−
Myalgia	+	++
Diarrhea	+	+
Headache	+	+
Arthralgia	−	++
Nausea	−	+
Rash	+/−	+/−
Respiratory support	++	+/−
Laboratory findings		
Leukopenia	+	−
Lymphocytosis	−	+
Lymphopenia	+	−
Thrombocytopenia	−	++
d-Dimer	++	+/−
Ferritin	++	+

It is important to keep in mind the clinical and epidemiological particularities of each virus in coinfection to better guide the diagnosis and prognosis. Thus, for example, dengue fever frequently has serious manifestations in young adults because most are experiencing the effects of a second infection, whereas COVID-19 is generally asymptomatic or has few symptoms in this population, as was observed in case 1. In the elderly, dengue severity depends mainly on the presence of comorbidities or the infectious serotype. However, COVID-19 is consistently more severe in older age groups, especially in patients with decompensated comorbidities, as was observed in the second case [11, 12].

In these cases, laboratory tests are very useful because, in addition to determining whether the agent is present in the body, tests help to discern the disease’s phase. However, it is important to consider tests’ limitations. Recently, the possible alteration of dengue serological test results during the COVID-19 pandemic has been reported. Yan *et al*. [5] described the case of two patients in whom SARS CoV-2 infection was confirmed by rRT-PCR, but in addition IgM/IgG serology was positive for dengue. However, RT-PCR for dengue was negative. Given the time of symptoms, the clinicians considered the dengue test to be a false-positive result [5].

According to this information, it is important to consider that, in primary dengue infection, both nonstructural protein 1 (NS1) and viral RNA can be detected from the onset of symptoms until approximately day 5 of infection. The immune response usually appears at two times. During the acute phase, immunoglobulin M (IgM) appears on approximately the third to fifth day of infection and may remain at detectable levels for several months. Immunoglobulin G (IgG) rises towards the end of the acute period, which lasts an average of 10 days and confers immunological memory for the infecting serotype, which can persist for years. IgG does not usually appear during the acute phase of primary disease. However, in secondary infection, IgG rises even earlier than IgM [13].

In SARS-CoV-2, the viral load in respiratory specimens is highest during the initial phases of the disease, reaching a peak during the second week of infection. It then decreases until it becomes undetectable in most cases. In severe disease, the viral load in respiratory fluids is highest at approximately the third and fourth weeks. The factors that cause the viral load to persist more in some individuals than in others remain to be clarified [14]. Synthesis of antibodies against SARS-CoV-2 is a primary immune response to infection [15]. IgM levels increase during the first week after infection, peaking after 2 weeks; they then tend to disappear in most individuals. IgG is detectable after the first week and remains elevated for approximately 90 days. However, it is not yet clear whether these antibodies can protect against reinfection during that time [16].

In summary, the negative result of the diagnostic tests, either in COVID-19 or dengue fever, does not rule out infections when the clinical symptoms are suggestive, especially in early stages. In patients with several days of symptoms, the probability of finding RT-PCR for dengue positivity is low, since the sensitivity of the test decreases further along the course of illness, as observed in the second case. It has been seen that RT-PCR tests for SARS CoV-2 can be positive for longer periods, which is an interesting difference compared with direct dengue tests [16, 17]. Given the above, the possibility of having discordant results in the diagnostic tests during dengue/SARS CoV-2 coinfection raises the need to clarify the dynamics of the clinical evolution of both diseases and to take positive or negative laboratory test results with caution [4, 18].

In conclusion, dengue virus and SARS CoV-2 coinfection are possible, and should be suspected in dengue endemic areas. The diagnosis requires a combination of tools, tests for the direct detection of virus, and indirect tests that measure the immune response, in addition to an adequate interpretation of the results according to the clinical and epidemiological particularities of both infections.

## Data Availability

The datasets used during the current study are available from the corresponding author on reasonable request.

## Abbreviations

WHO: World Health Organization
AST: Aspartate aminotransferase
ALT: Alanine aminotransferase
WBC: White blood cell
ICU: Intensive care unit
